# Investigating the role of the TGF-β–SLC20A1 axis in the spatial heterogeneity of hepatocellular carcinoma through single-cell and spatial transcriptomics

**DOI:** 10.3389/fimmu.2026.1723334

**Published:** 2026-03-24

**Authors:** Jun Li, Jingqi An, Siyuan Jiang, Xuyang Wang, Fei Zhang, Jinfei Liu, Wenbin Li, Mengdi Wang, Xinjun Wu, Shuangshuang Li, Weilin Wang, Tao Yu, Xing Liu, Meng Li

**Affiliations:** 1 Department of Hepatobiliary Pancreatic Splenic Surgery, Fuyang Women and Children’s Hospital, Fuyang, China; 2 West China Hospital, Sichuan University, Chengdu, Sichuan, China; 3 First Clinical Medical College (First Affiliated Hospital), Anhui Medical University, Hefei, China; 4 Department of Anorectal Surgery, Fuyang Women and Children’s Hospital, Fuyang, China

**Keywords:** hepatocellular carcinoma, single-cell RNA sequencing, SLC20A1, spatial transcriptomics, TGF-β signaling pathway

## Abstract

**Background:**

Hepatocellular carcinoma (HCC) is a highly heterogeneous malignancy characterized by marked cellular and spatial diversity within the tumor microenvironment (TME). The transforming growth factor-β (TGF-β) signaling pathway plays a dual role in the initiation and progression of HCC. However, the spatial distribution characteristics and key regulatory mechanisms of TGF-β signaling within HCC tissues remain inadequately elucidated.

**Methods:**

This study integrated spatial transcriptomics (ST), single-cell RNA sequencing (scRNA-seq), and bulk RNA-seq data to systematically characterize the spatial heterogeneity of the TGF-β signaling pathway in HCC. By combining non-negative matrix factorization (NMF), CellChat-based cell–cell communication analysis, and multi-algorithm machine learning approaches, we identified key driver genes closely associated with TGF-β activity. Subsequently, CCK-8 assays, colony formation, wound-healing, and Western blot experiments were performed in HCC cell lines to validate the biological functions of the identified gene.

**Results:**

The results revealed that the TGF-β signaling pathway exhibited the highest activity at the tumor–stroma interface, which was enriched with cancer-associated fibroblasts (CAFs), immunosuppressive cells, and genes related to extracellular matrix (ECM) remodeling. CellChat analysis showed that TGF-β–TGFBR ligand–receptor interactions between tumor cells, CAFs, and immune cells were markedly enhanced, contributing to the formation of a localized immunosuppressive microenvironment. Machine learning analysis identified SLC20A1 as a key regulatory factor. Functional assays demonstrated that SLC20A1 enhances the proliferation, migration, and epithelial–mesenchymal transition (EMT) of HCC cells, whereas its knockout significantly suppresses these malignant phenotypes.

**Conclusion:**

This study represents the first comprehensive integration of spatial and single-cell transcriptomics to uncover the spatial organization of TGF-β signaling in HCC and to identify the TGF-β–SLC20A1 axis as a critical driver of tumor invasion at the tumor–stroma interface. Our findings provide new mechanistic insights into tumor–stroma interactions and suggest a potential therapeutic target for precision treatment of HCC.

## 1 Introduction

Hepatocellular carcinoma (HCC) is the most prevalent primary liver malignancy worldwide, and its high invasiveness and propensity for metastasis severely compromise patient survival. According to the Global Cancer Observatory, HCC ranked sixth in global cancer incidence and third in cancer-related mortality in 2022, and its incidence is projected to continue rising over the next three decades (1, 2). Despite advances in multimodal therapies—including surgical resection, local ablation, molecularly targeted agents, and immune checkpoint inhibitors—most patients are diagnosed at intermediate or advanced stages, and their prognosis remains unsatisfactory. As a prototypical inflammation-driven tumor, the development of HCC is closely associated with chronic hepatitis, fatty liver disease, cirrhosis, and immune dysregulation (3, 4). Elucidating the functions and interactions of diverse cellular subpopulations within the tumor microenvironment (TME) is critical for uncovering the mechanisms underlying HCC initiation and progression and for identifying novel therapeutic strategies.

With the rapid advancement of single-cell RNA sequencing (scRNA-seq) and spatial transcriptomics (ST) technologies, it has become evident that HCC exhibits not only pronounced genetic and phenotypic heterogeneity but also distinct spatial compartmentalization. For example, Zhang et al. employed ST analysis and found that the tumor–interface region was enriched with fibroblasts and immunosuppressive myeloid cells, accompanied by upregulation of genes associated with extracellular matrix remodeling (5). Similarly, Gu et al. demonstrated through ST profiling that proliferative signaling of tumor cells was markedly enhanced in the tumor core, whereas the interface region displayed a higher density of immune cell infiltration (6). These findings highlight significant differences among the tumor core, tumor–interface, and adjacent non-tumor regions in terms of cellular composition, signaling pathway activity, metabolic state, and immune cell infiltration. Such spatial heterogeneity is often closely linked to tumor aggressiveness, immune evasion, and therapeutic response. However, although spatial transcriptomic and single-cell studies have provided valuable insights into the spatial distribution of cell types and microenvironmental features in HCC, systematic investigations of signaling pathway activities and their regulatory mechanisms across different spatial regions remain limited, highlighting the need for spatially resolved pathway-level analyses.

The TGF-β signaling pathway is one of the key drivers of HCC progression (7). In the early stages of the disease, this pathway exerts tumor-suppressive effects; however, when persistently activated during tumor progression, it induces epithelial-to-mesenchymal transition (EMT), promotes extracellular matrix deposition, and enhances immunosuppression, thereby accelerating tumor cell migration, invasion, and metastasis (8, 9). Studies have revealed pronounced spatial heterogeneity of TGF-β signaling in HCC, with the tumor–interface region frequently exhibiting the highest activity, which is strongly associated with tumor cell infiltration, angiogenesis, and poor clinical outcomes (10). Nevertheless, the key downstream effectors shaping the spatial activity of the TGF-β signaling pathway and their roles in mediating tumor–stroma interactions remain insufficiently elucidated, and a systematic, integrative characterization of its spatial activity landscape at single-cell resolution in HCC is still lacking.

In this study, we integrated spatial transcriptomic and single-cell transcriptomic data, combined with non-negative matrix factorization (NMF), cell–cell communication analysis, and machine learning approaches, to systematically characterize the activity landscape of the TGF-β pathway across distinct spatial regions and cellular subpopulations in HCC, and identified key regulatory factors closely associated with tumor progression. We further validated their roles in modulating tumor cell phenotypes through *in vitro* functional assays. This study aims to elucidate the TGF-β-driven spatial heterogeneity of HCC and its underlying mechanisms, thereby providing a theoretical basis for advancing the understanding of liver cancer progression and developing novel precision therapeutic strategies.

## 2 Materials and methods

### 2.1 Study design

**Figure 1:**
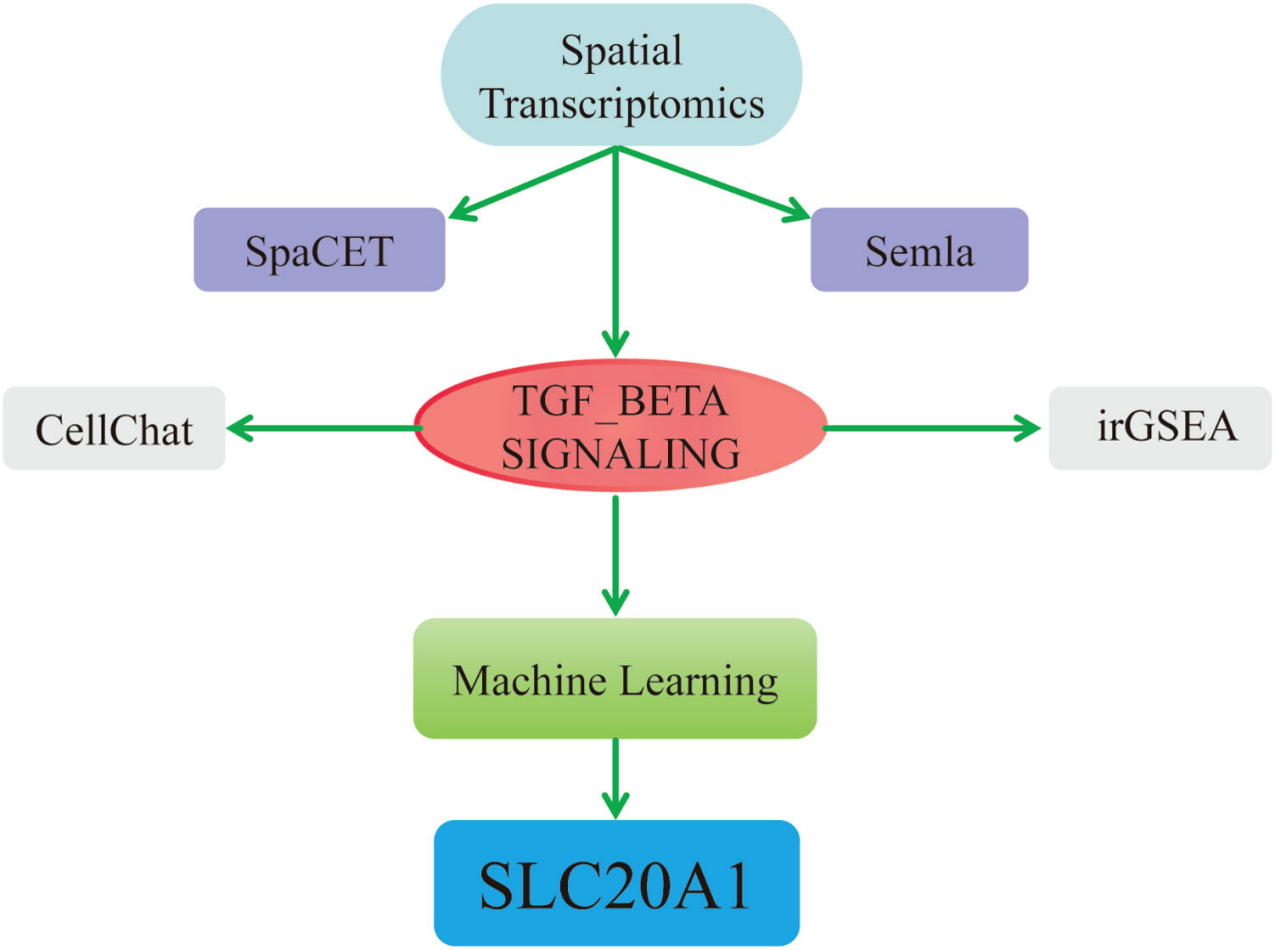
Flowchart of the analysis.

The workflow of this study is depicted in **Figure 1**. This study employed an integrated multi-omics and machine learning approach, combining spatial transcriptomics, single-cell sequencing, and RNA-seq analyses to identify SLC20A1 as a novel key gene in hepatocellular carcinoma and establish its association with the dysregulated TGF-β signaling pathway.

### 2.2 Data sources

All data supporting the findings of this study are publicly available from The Cancer Genome Atlas (TCGA) and the NCBI Gene Expression Omnibus (GEO). Single-cell RNA sequencing (scRNA-seq) and spatial transcriptomics (ST) data, along with associated images, were retrieved from the GEO datasets GSE235057 and GSE245908. Furthermore, data from the Cancer Dependency Map (DepMap) project were utilized for SLC20A1 *in vitro* validation of genetic dependencies (11). Additional details regarding data sources are included in the article.

### 2.3 Processing of scRNA-seq data

We processed scRNA-seq data from 10 samples with Liver cancer using the Seurat package (12). The samples were merged into a single Seurat object, and the percentage of mitochondrial, ribosomal, and hemoglobin genes was calculated for quality control. Cells were retained if they met all of the following criteria: percent_mito < 25, percent_ribo > 3, and percent_hb < 1. Cells were filtered based on these metrics. Following data normalization, we identified and scaled highly variable genes and performed principal component analysis (PCA). Batch effects were corrected on the principal components using Harmony, with the optimal dimensionality for downstream analysis determined from an elbow plot. A nearest-neighbor graph was constructed and clustered at a resolution of 1.0, following which the results were visualized using UMAP.

To accurately annotate cell types, we first applied the FindAllMarkers function to identify significantly upregulated genes in each cluster, using thresholds of >25% gene expression and a log2 fold change >0.25. The top 30 marker genes with the highest average log2 fold change were selected from each cluster and submitted to the CellMarker 2.0 database for annotation (13). Next, we employed the SingleR package with the celldex reference dataset for major cell group annotation, deriving predictions from both broad and refined labels (14). Finally, cells were manually reassigned based on their annotated types, and these results were incorporated into the Seurat object’s metadata for downstream analysis.

### 2.4 Processing and analysis of spatial transcriptomics data

The 10x Genomics spatial transcriptomics dataset was processed using the Seurat package. Data were normalized with SCTransform while retaining all genes, followed by dimensionality reduction via principal component analysis (PCA). Cell clustering was performed using a nearest-neighbor algorithm applied to the principal components and visualized with UMAP. Spatial transcriptomics sample was deconvoluted, subjected to spatial NMF clustering, and assessed for tumor boundaries using the SpaCET and Semla R packages (15, 16).

### 2.5 Analysis of scRNA-seq data

To analyze intercellular communication from scRNA-seq data, a CellChat object was created with cells stratified by their pathway activity scores, using the human CellChat database as a reference (17). Gene set enrichment analysis was performed using the irGSEA R package to investigate the functional state heterogeneity across different cell types. Enrichment scores for gene sets from the Hallmark collection were calculated for each cell using the irGSEA.score function with the 6 different method (18).

### 2.6 Machine learning based feature selection

Feature gene selection was performed on the TCGA-LIHC transcriptomic dataset using an ensemble of five machine learning methods to ensure robust identification of prognostic features. The methods included Step-back, Random Forest (RF), glmboost, step_both, and Lasso regression. All models were tuned with standard procedures in randomForest, mboost, caret and MASS R packages. A gene was considered a high-confidence feature if it was selected by all of the five employed methods, thereby minimizing model-specific bias and enhancing the biological reliability of the findings.

### 2.7 Cell culture and lentiviral infections

This study utilized human hepatocellular carcinoma cell lines MHCC97H and Hep3B, which were cultured in Dulbecco’s Modified Eagle Medium (DMEM) supplemented with 10% fetal bovine serum (FBS) at 37 °C in a 5% CO_2_ incubator. To establish stable SLC20A1-knockout and overexpression models, cells were infected with lentiviral particles at approximately 50–60% confluence. Specifically, gene knockout was achieved using a lentiviral CRISPR/Cas9 system expressing a target-specific sgRNA (5′-GACATGAAACCAGACAACAG-3′), while overexpression was performed using a commercially synthesized SLC20A1 full-length cDNA clone packaged into a lentiviral vector. After puromycin selection, Western blot analysis confirmed the successful manipulation of SLC20A1 expression in each cell model. Functional assays revealed that SLC20A1 knockout significantly suppressed the proliferation, migration, and invasion of hepatocellular carcinoma cells, whereas its overexpression markedly enhanced these malignant phenotypes. Collectively, these results substantiate the promotive role of SLC20A1 in hepatocellular carcinoma progression.

### 2.8 Western blot

A freshly prepared radioimmunoprecipitation assay (RIPA) buffer containing phenylmethylsulphonyl fluoride (PMSF) at a 100:1 ratio was used for tissue homogenization. The tissue fragments were lysed using a mechanical homogenizer followed by centrifugation at 12,000 rpm for 30 minutes at 4 °C. The resulting supernatant was collected and mixed with protein loading buffer at a 4:1 (v/v) ratio, then denatured by boiling at 100 °C for 10 minutes. Protein separation was performed using a PAGE Gel Fast Preparation Kit, and the resolved proteins were transferred onto a polyvinylidene fluoride (PVDF) membrane. The membrane was successively incubated with primary antibodies at 4°C overnight and corresponding secondary antibodies for 2 hours at room temperature, with three washes of TBST (Tris-buffered saline with 0.1% Tween^®^ 20) between incubations. Protein bands were visualized using enhanced chemiluminescence, and their intensity was quantified with ImageJ software.

### 2.9 Cell counting kit-8

Cells were plated in 96-well plates at a density of 1,000 cells per well and subjected to the indicated treatments. Following incubation at 37 °C under 5% CO_2_, 10 μL of CCK-8 solution was added to each well at the designated time points. The plates were further incubated under the same conditions, and the absorbance at 450 nm was measured.

### 2.10 Colony formation assays

Cells were seeded in 60 mm culture dishes at a density of approximately 1,500 cells per dish and maintained in a 37 °C, 5% CO_2_ incubator. Upon the formation of microscopically visible colonies of appropriate size and number, the cultures were processed as follows: dishes were rinsed with PBS, fixed with 4% polymethanol for 20 minutes, and then stained with Giemsa solution for 30 minutes. After staining, the dishes were rinsed under running tap water, air-dried, photographed, and the colonies were quantified.

### 2.11 Wound healing assays

We seeded cells in 6-well plates and allowed them to form a confluent monolayer. Using a sterile pipette tip guided by a straightedge, we generated a linear scratch across the cell monolayer. After washing three times with PBS to remove debris, we added serum-free medium and incubated the cells. Migration into the wound area was documented at 0 h and 24 h using a 10x microscope objective.

### 2.12 Transwell

To evaluate the role of the TGF-β-SLC20A1 axis in hepatocellular carcinoma (HCC) cell migration, Transwell migration assays were performed using Hep3B and MHCC-97H cells. For the assay, 1–5 × 10^4^ cells (depending on cell line optimization) in serum-free medium were seeded into the upper chamber of 8-μm pore Transwell inserts (Corning), while the lower chamber contained serum-free medium supplemented with either recombinant human TGF-β (5 ng/mL) or an equivalent volume of IgG isotype control. Following incubation at 37 °C with 5% CO_2_ for 24–48 h (optimized per cell line), non-migrated cells on the upper surface were gently removed with a cotton swab, and migrated cells on the lower surface were fixed with 4% paraformaldehyde, stained with crystal violet (0.1–0.5%), and quantified by counting five random fields under a light microscope or by image analysis software. Each condition was performed in triplicate, and experiments were repeated at least three times independently.

## 3 Result

### 3.1 Spatial transcriptomics analysis with SpaCET

**Figure 2:**
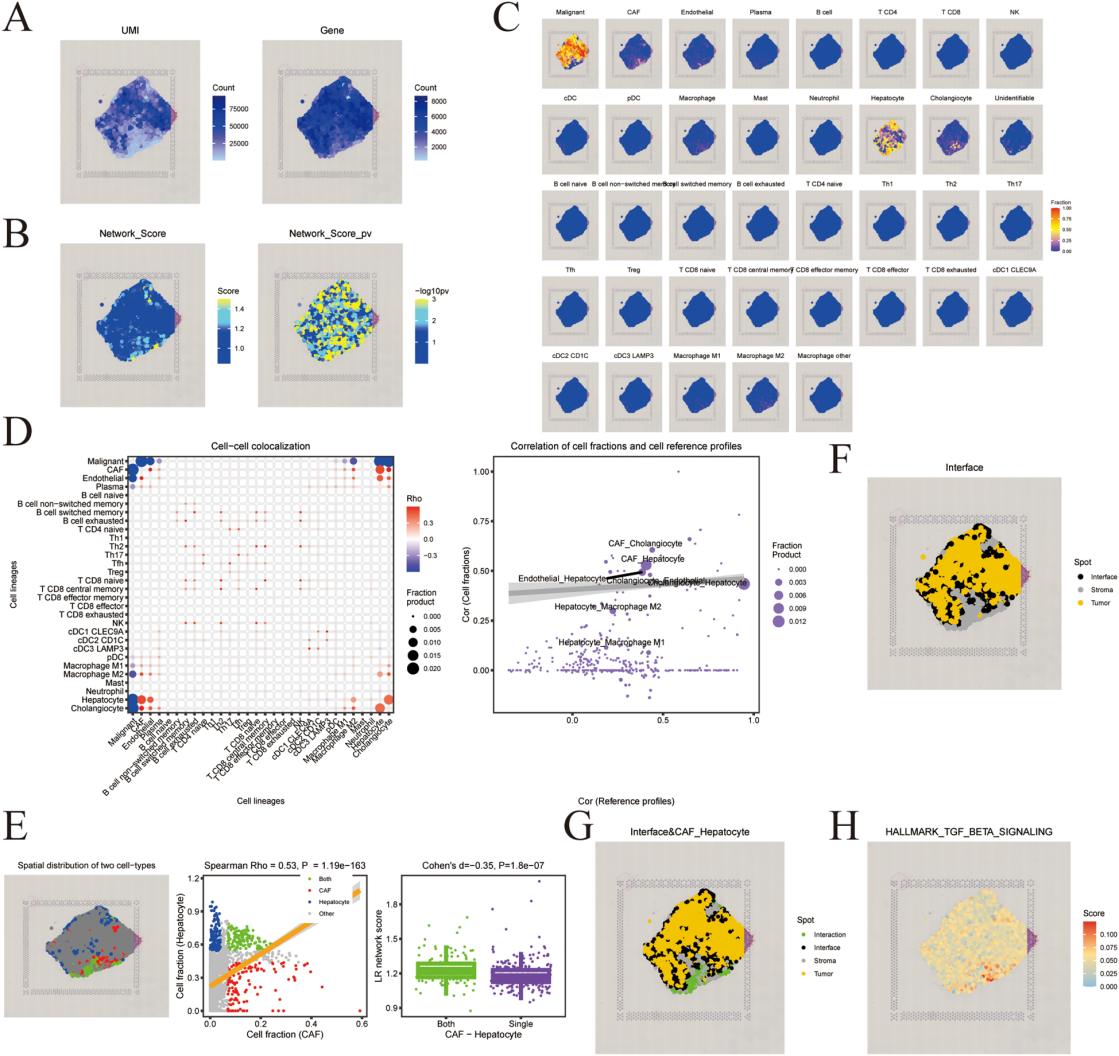
**(A)** Filter spatial spots and calculate the QC metrics. **(B)** Spatial feature visualization of Network_Score and Network_Score_pv **(C)** Spatial feature visualization of deconvolution result **(D)** Cell-cell colocalization analysis of spatial analysis. **(E)** Ligand-Receptor analysis for a co-localized cell-type pair. **(F)** Identify tumor-stroma interface. **(G)** Tumor-stroma interface and CAF_Hepatocyte interaction visualization. **(H)** HALLMARK_TGF_BETA_SIGNALING expression in spatial spots.

We analyzed spatial transcriptomics samples using the SpaCET framework. The data quality of the spatial transcriptomics samples and the quality of the network construction are illustrated in **Figures 2A, B**, respectively. Leveraging established immune cell and HCC marker genes, we deconvoluted the data to identify malignant regions and spatially map the infiltration patterns of cell types in **Figure 2C**. The correlations among cell type proportions derived from the deconvolution analysis are presented in **Figure 2D**. Cancer-associated fibroblasts (CAFs) and hepatocytes demonstrated a strong positive correlation, with additionally pronounced interactions observed within the stromal compartment in **Figures 2E–G**. Notably, we concurrently observed a marked enrichment of the TGF-β signaling pathway in these same regions in **Figure 2H**. This finding suggests the potential involvement of the TGF-β signaling pathway in this underlying oncogenic process.

### 3.2 Spatial transcriptomics analysis with Semla

**Figure 3:**
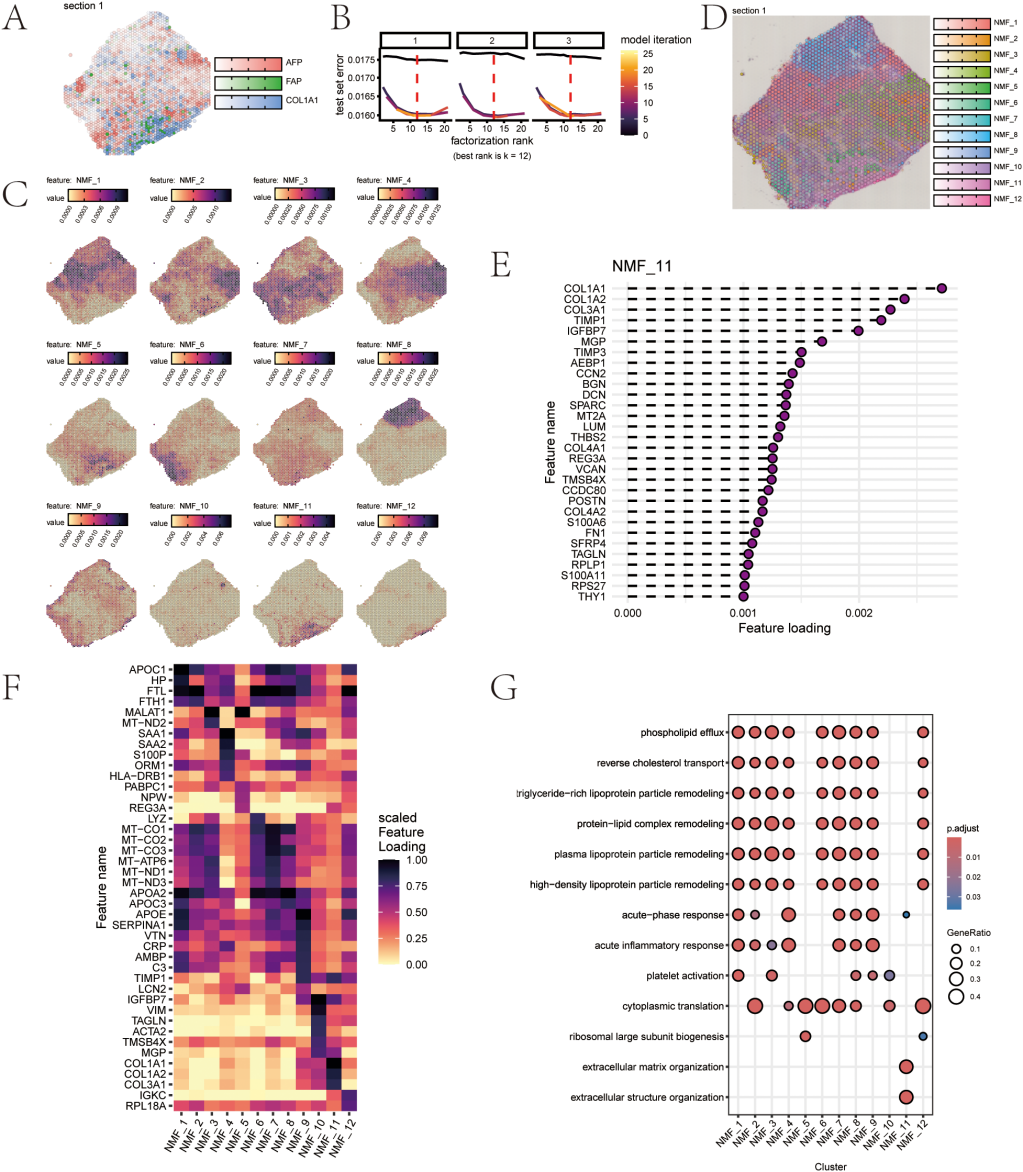
**(A)** Visualization of categorical features of area. **(B)** NMF rank selection evaluation plot. **(C)** NMF Features visualization in spatial spots separately. **(D)** NMF Features visualization in spatial spots holistically. **(E)** Top gene contribution in NMF_11 **(F)** Heatmap for NMF factors. **(G)** Enrichment for NMF factors.

The spatial projection of AFP, FAP, and COL1A1 in the spatial transcriptomics samples is shown in **Figure 3A**, which confirms the presence of the stromal region. The cross-validation results indicated an optimal value at k=12 shown in **Figure 3B**. The spatial Unsupervised non-negative matrix factorization (NMF) clustering analysis yielded twelve distinct clusters, as shown in **Figures 3C, D**. NMF_11, identified as the module of primary interest in our study, exhibits its gene loadings as shown in **Figure 3E**. The gene contribution heatmap along with the functional enrichment analysis results are presented in **Figures 3F, G**. Extracellular matrix organization and extracellular structure organization pathways were enriched in NMF_11, which creates favorable conditions for tumor growth, invasion, and metastasis.

### 3.3 scRNA-seq analysis in liver cancer

**Figure 4:**
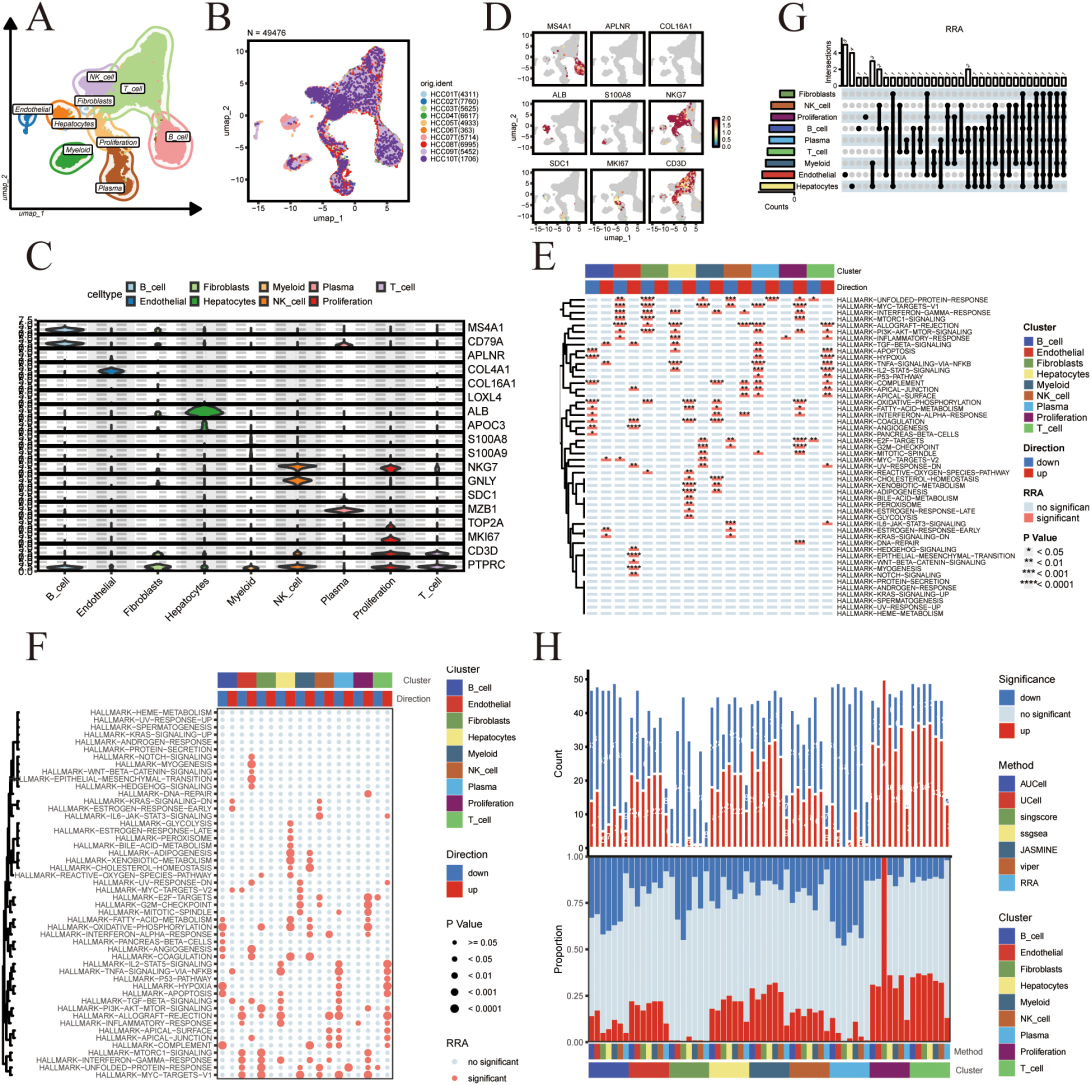
**(A)** UMAP projection of single-cell celltype. **(B)** UMAP projection of single-cell samples **(C)** Violin plot displaying marker gene expression for the 9 distinct cell types. **(D)** Feature Plots for celltype markers. **(E)** Heatmap shows co-upregulated or co-downregulated gene sets per cluster in RRA. **(F)** Bubble plot shows co-upregulated or co-downregulated gene sets per cluster in RRA. **(G)** Upset plot show the intersections of significant gene sets among clusters in RRA. **(H)** Stacked bar plot shows the intersections of significant gene sets among clusters in all methods.

ScRNA-seq data were gathered from 10 superficial liver cancer samples. After rigorous analysis, nine major cell types were identified including B cell, T cell, NK cell, Fibroblast, Proliferation, Myeloid, Hepatocytes, Endothelial and Plasma in **Figures 4A, B**. The violin plot and the projection of marker genes on UMAP are shown in **Figures 4C, D**. Heatmap and Bubbleplot Show co-upregulated or co-downregulated gene sets per cluster in RRA in **Figures 4E, F**. Upset plot and stacked bar plot show the intersections of significant gene sets among clusters in all methods in **Figures 4G, H**.

### 3.4 Characteristics of TGF-β signaling pathway

**Figure 5:**
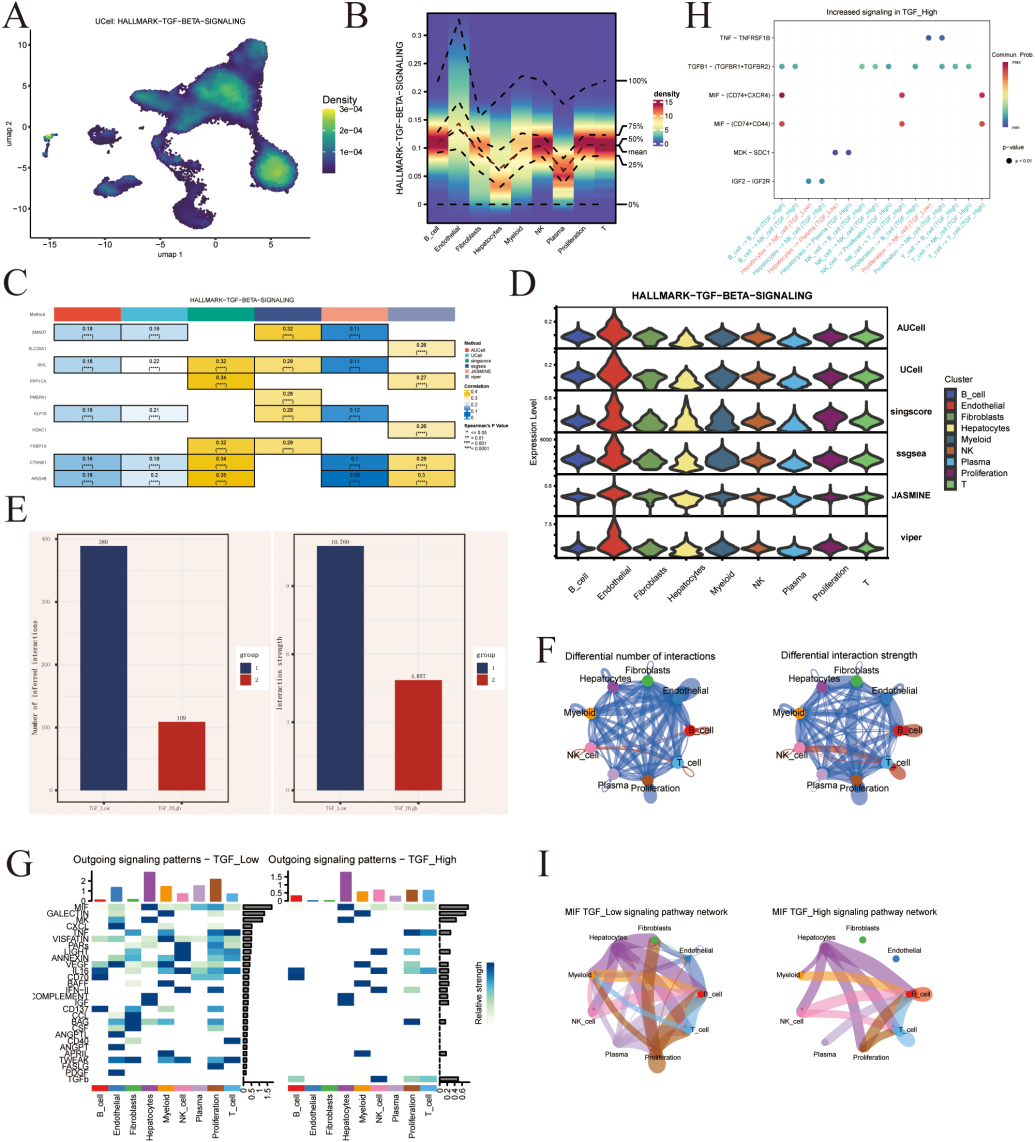
**(A)** UMAP projection of HALLMARK_TGF_BETA_SIGNALING. **(B)** Density plot of HALLMARK_TGF_BETA_SIGNALING. **(C)** Hub gene of TGF geneset. **(D)** Vlnplot show the expression and distribution of TGF pathway between AUCell, UCell, singscore, ssgsea, JASMINE and viper among clusters. **(E)** Number of inferred interactions and interaction strength between TGF low and high group. **(F)** Differential number of interactions and Differential interaction strength. **(G)** Heatmap of Outgoing in TGF low and high. **(H)** Increased signaling in TGF_High. **(I)** MIF in TGF_Low signaling pathway network and TGF_High signaling pathway network.

The enrichment of the TGF-β signaling pathway projected onto the UMAP is presented in **Figure 5A** using Nebulosa method in UCell. Expression of the TGF-β signaling pathway across different cell types is shown in **Figures 5B, D**. The hub genes of the TGF-β signaling pathway were identified based on the correlation between the TGF-β pathway activity score and the expression levels or ranks of genes within the gene set, as shown in **Figure 5C**, with SLC20A1 being identified as a key gene. We subsequently stratified the cells into two groups based on the activity level of the TGF-β signaling pathway and performed CellChat analysis to investigate the alterations in underlying signaling pathways. Different number of interaction and interaction strength were shown in **Figures 5E, F**. Increased signaling in TGF_High group was shown in **Figure 5H**, which MIF exhibits a significant difference between the high and low groups in **Figure 5I**. Heatmap of outgoing signaling patterns was shown in **Figure 5G** between Low and High group.

### 3.5 Identification of key genes SLC20A1

**Figure 6:**
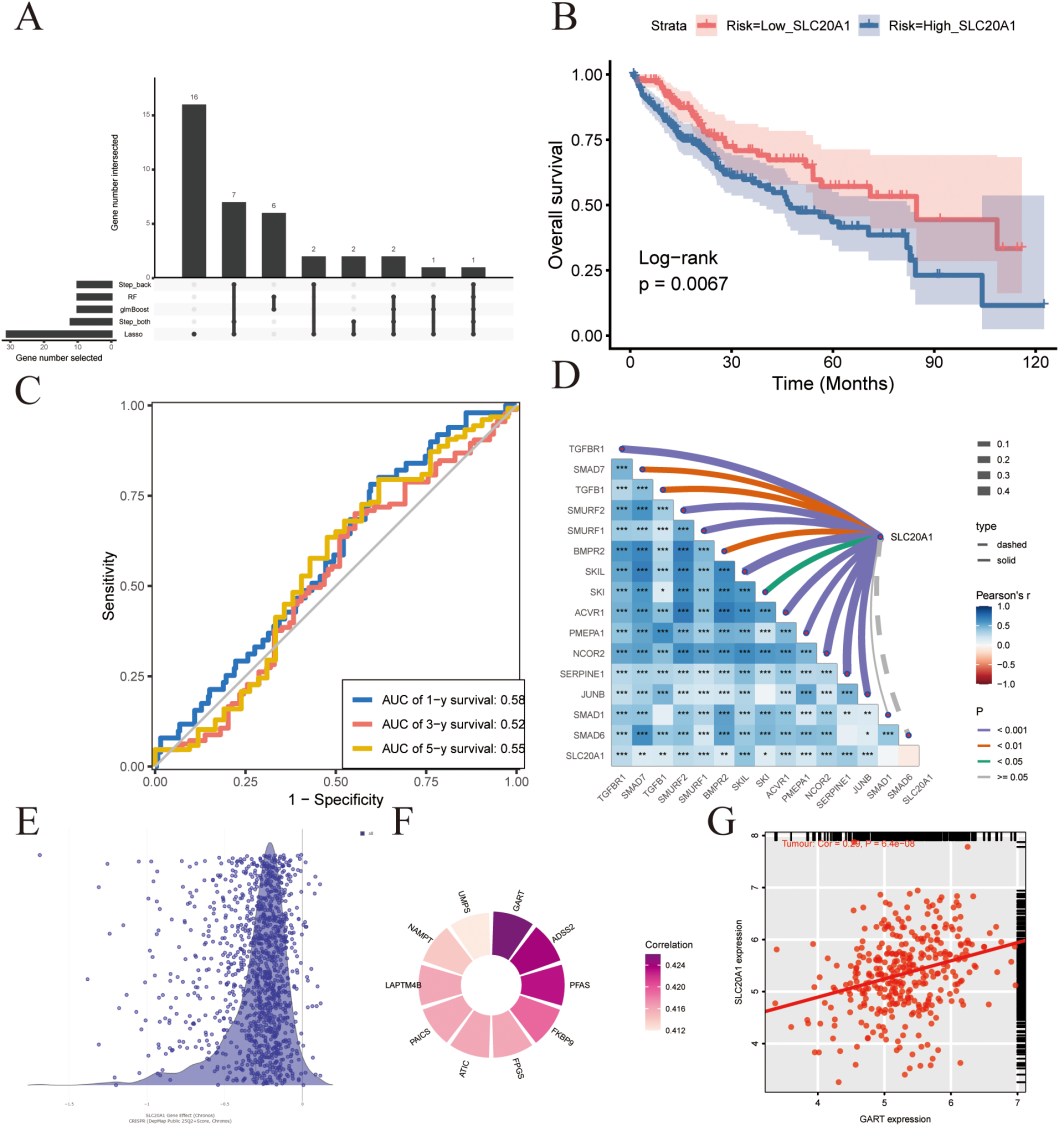
**(A)** Upset plot of 5 Machine Learning. **(B)** KM plot for SLC20A1 in TCGA-LIHC. **(C)** ROC plot for SLC20A1 in 1, 3, 5years. **(D)** Cor plot for SLC20A1 with TGF genes. **(E)** DepMap analysis for SLC20A1. **(F)** SLC20A1-Associated Genes in DepMap. **(G)** Cor plot between SLC20A1 and GART. Statistical significance was indicated as follows: * P < 0.05, ** P < 0.01, and *** P < 0.001.

Using a set of feature selection techniques including Step-back, Random Forest (RF), glmboost, step_both, and Lasso regression, key genes were identified. SLC20A1 was consistently pinpointed as a member of the TGF-β signaling pathway by all five algorithms in **Figure 6A**. The Kaplan-Meier curve demonstrated a trend toward prognostic significance for SLC20A1 (p=0.067) in **Figure 6B**, while the ROC curve for the single gene SLC20A1 also demonstrated predictive value in **Figure 6C**. To further investigate the association between SLC20A1 and pathway genes, we performed a correlation analysis, and the results are shown in **Figure 6D**. As shown in the Depmap data, knockdown of SLC20A1 attenuated cellular proliferative and migratory capacities in **Figure 6E**. The genes most correlated with SLC20A1 were identified, among which GART was determined to be positively correlated with SLC20A1 in **Figures 6F, G**.

### 3.6 Functional characterization of SLC20A1 in oncogenic phenotypes using gain- and loss-of-function studies

**Figure 7:**
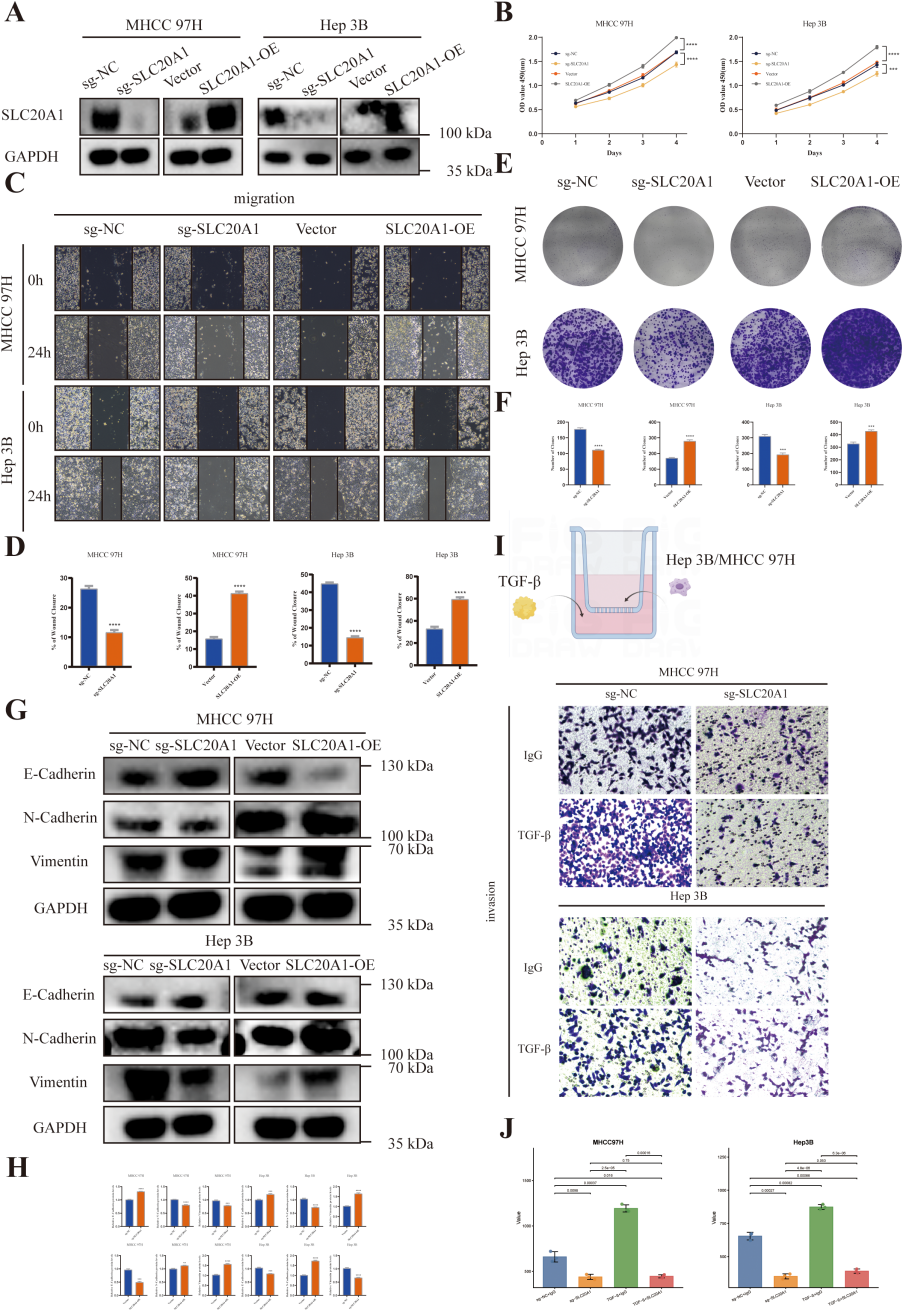
**(A)** Western blot validation of SLC20A1 knockout and overexpression in Hep3B and MHCC97H cell lines. **(B)** Cell growth rates of the indicated cells were evaluated by CCK-8 assays. **(C, D)** Wound healing assays. **(E, F)** Colony formation assays. **(G, H)** The expression of EMT markers (E-cadherin, N cadherin and Vimentin) was detected by Western blot analysis. **(I, J)** Transwell invasion assays of MHCC97H and Hep3B cells with sg-NC or sg-SLC20A1 under IgG or TGF-β treatment. Statistical significance was indicated as follows: ** P < 0.01, *** P < 0.001, and **** P < 0.0001.

To investigate the biological functions of SLC20A1 in HCC, we established stable knockout and overexpression cell lines. Transfection efficiency was confirmed by western blotting as shown in **Figure 7A**. To evaluate the proliferative capacity of HCC cells, CCK-8 and colony formation assays were employed. The results indicated that SLC20A1 knockout significantly attenuated both cell viability and clonogenic potential in MHCC97H and Hep3B cell lines in **Figures 7B, E**. Furthermore, to determine the role of SLC20A1 in metastatic behaviors, wound healing was conducted. This analysis revealed that SLC20A1 depletion markedly suppressed the migratory and invasive capabilities of the cells in **Figure 7C**. Furthermore, Western blot analysis revealed that SLC20A1 knockout in MHCC97H and Hep3B cells led to a marked upregulation of E-cadherin and a concomitant downregulation of vimentin and N-cadherin in **Figure 7G**, a pattern consistent with the suppression of epithelial-mesenchymal transition (EMT).

### 3.7 The TGF-β-SLC20A1 axis promotes tumor cell migration

Knockout of SLC20A1 significantly suppressed the basal migratory capacity of both Hep3B and MHCC-97H cells in Transwell assays compared to sg-NC controls (P < 0.01). Exogenous addition of recombinant TGF-β to the lower chamber markedly promoted migration of sg-NC cells in both cell lines, consistent with its established role as a pro-migratory factor in HCC. However, this TGF-β-induced enhancement of migration was completely abolished in sg-SLC20A1 cells, indicating that SLC20A1 is required for mediating the pro-migratory effects of TGF-β. These findings demonstrate that the TGF-β-SLC20A1 axis plays a critical role in regulating HCC cell migration, with SLC20A1 acting as an essential downstream mediator of TGF-β signaling in this context (**Figures 7I, J**).

## 4 Discussion

HCC is a highly heterogeneous malignancy whose initiation and progression are driven not only by intrinsic genetic alterations within tumor cells but also by the remodeling of stromal cells, immune components, and signaling pathways in the tumor microenvironment. Recent advances in single-cell and spatial transcriptomic technologies have enabled the dissection of tumor spatial organization and regulatory mechanisms at tissue-context resolution. In this study, we integrated spatial transcriptomic and single-cell transcriptomic datasets and applied non-negative matrix factorization (NMF), cell–cell communication analysis, and machine-learning approaches to systematically delineate the spatial activity landscape of the TGF-β signaling pathway in HCC and identified SLC20A1 as a key molecule associated with invasive characteristics at the tumor–stroma interface. Furthermore, *in vitro* functional assays confirmed the role of SLC20A1 in modulating tumor cell phenotypes. These findings provide new insights into the spatial heterogeneity of HCC and offer a basis for exploring potential therapeutic targets.

This study first revealed that the TGF-β signaling pathway exhibits the highest activity at the tumor–stroma interface. Compared with the tumor core, the interface region harbors a more complex cellular composition, characterized not only by infiltrating hepatocytes but also by an enrichment of cancer-associated fibroblasts (CAFs), immunosuppressive myeloid cells, and a subset of functionally exhausted CD8^+^ T cells. Spatial transcriptomic analysis demonstrated that the distribution of these cells coincides with high expression of genes involved in extracellular matrix (ECM) remodeling, suggesting that the interface region establishes a distinctive microenvironment defined by ECM reconstruction and immunosuppression. This observation is consistent with the perspective proposed by Hanahan and colleagues, who highlighted that CAFs remodel the tumor stroma through secretion of ECM components such as collagen and fibronectin, and, via factors including TGF-β and PGE_2_, recruit immunosuppressive myeloid cells and dampen T-cell activity, thereby fostering an immunosuppressive niche (19). Likewise, Massagué and co-workers reported that aberrant activation of TGF-β signaling in cancer promotes ECM remodeling, suppresses effector T-cell responses, and enhances the activity of immunosuppressive cells, collectively driving tumor progression and immune evasion (20). These findings align with our observations of heightened TGF-β activity, CAF accumulation, and an immunosuppressive niche at the HCC interface, suggesting that spatially confined activation of TGF-β signaling may orchestrate CAF activation and ECM remodeling, thereby shaping an immune-suppressive microenvironment that facilitates tumor invasion and immune escape.

Secondly, we identified SLC20A1 as a key molecule closely associated with the spatial activity of the TGF-β signaling pathway. SLC20A1 encodes a type III sodium-dependent inorganic phosphate transporter (PiT-1), initially recognized for mediating transmembrane transport of extracellular inorganic phosphate (Pi), but later found to exert transport-independent regulatory functions in cell signaling (21, 22). In mice, SLC20A1 has been shown to be essential for liver development, underscoring its biological significance in hepatic tissue (23). In cancer, aberrant SLC20A1 expression has been reported to promote cell proliferation, migration, and invasion; for example, in head and neck squamous cell carcinoma (HNSCC), high SLC20A1 expression correlates with poor prognosis (24), while in hepatic cell models, SLC20A1 knockout affects signaling molecules such as USP7, implicating it in metabolic–signaling regulation (25). In the present study, we further validated the pro-invasive role of SLC20A1 in HCC cells through *in vitro* functional assays: silencing SLC20A1 markedly inhibited wound-healing migration and Matrigel invasion, accompanied by a reversal of canonical epithelial-to-mesenchymal transition (EMT) markers—upregulation of E-cadherin and downregulation of N-cadherin and Vimentin—whereas overexpression of SLC20A1 promoted faster migration, stronger invasive capacity, and enhanced EMT features. These findings suggest that SLC20A1 functions not merely as a phosphate transporter but as a signaling regulatory node that promotes EMT and invasion in HCC cells, potentially by modulating TGF-β-related signaling networks to drive the EMT process and thereby enhance the migratory and invasive capabilities of HCC cells.

Moreover, cell–cell communication analysis revealed a marked enrichment of TGF-β–TGFBR ligand–receptor interactions between tumor cells at the interface and CAFs as well as immune cells, accompanied by heightened activity of the ECM–receptor signaling pathway. This pattern is consistent with the protumorigenic role of TGF-β in advanced cancers, where it drives stromal remodeling, immunosuppression, and invasive phenotypes. TGF-β can activate fibroblasts and promote extracellular matrix (ECM) deposition while suppressing effector T-cell responses and enhancing the activity of immunosuppressive cells, thereby establishing a microenvironment at the tumor margin that favors invasion and immune evasion (20, 26). Other spatial-omics studies have likewise shown that the HCC interface is the most active region of cell–stroma–immune interactions, locally enriched with fibroblasts, immunosuppressive myeloid cells, and stressed hepatocytes, and characterized by prominent ECM remodeling and restricted T-cell function (27, 28). In addition, activation of the ECM–receptor pathway is often accompanied by increased matrix stiffness and induction of the epithelial-to-mesenchymal transition (EMT) program; in HCC, higher stromal stiffness has been shown to promote EMT and enhance invasion and metastasis (29). Against this background, we further found that high SLC20A1 expression is associated with ECM remodeling and an immunosuppressive phenotype, suggesting that it may represent a potential target for molecular intervention specifically at the tumor–stroma interface. Although the direct role of SLC20A1 in modulating TGF-β signaling remains to be fully elucidated, this finding provides a new candidate factor for uncovering the molecular mechanisms by which the interface microenvironment drives tumor progression.

Despite the novel insights gained in this study, several limitations should be acknowledged. First, the spatial and single-cell transcriptomic datasets analyzed were limited in sample size and were predominantly derived from advanced-stage HCC, which may not fully represent early-stage disease or the full spectrum of molecular subtypes. Second, although integrative multi-omics analyses and *in vitro* functional assays consistently highlighted the involvement of the TGF-β–SLC20A1 axis in HCC progression, these findings primarily indicate strong associations rather than definitive causal relationships. Further mechanistic experiments are therefore required to establish a direct regulatory link between TGF-β signaling and SLC20A1, particularly *in vivo* and in clinical settings. In addition, the functional validation relied mainly on *in vitro* experiments and lacked confirmation from animal models and patient-derived specimens. Future studies integrating larger-scale multi-omics cohorts, high-resolution spatial multi-omics technologies, and *in vivo* models will be essential to elucidate the dynamic and causal roles of the TGF-β–SLC20A1 axis across different disease stages and spatial contexts, and to evaluate its translational potential as a therapeutic target in HCC.

## 5 Conclusions

This study, based on an integrative multi-omics analytical framework, combined spatial transcriptomics, single-cell transcriptomics, and bulk RNA-seq data to systematically elucidate the spatial heterogeneity of the TGF-β signaling pathway in HCC. The results revealed that TGF-β signaling exhibited the highest activity at the tumor–stroma interface, which was enriched with cancer-associated fibroblasts (CAFs) and immunosuppressive cells, forming a localized pro-tumorigenic microenvironment. Through machine learning–based feature selection, SLC20A1 was identified as a key regulatory gene closely associated with TGF-β pathway activity. Functional experiments further confirmed that SLC20A1 promotes the proliferation, migration, and epithelial–mesenchymal transition (EMT) of HCC cells, while its knockout significantly suppresses these malignant phenotypes. In conclusion, this study reveals the central role of the TGF-β–SLC20A1 axis in spatial remodeling and progression of HCC, and proposes SLC20A1 as a promising potential target for disrupting tumor–stroma interactions and advancing precision therapy in HCC.

## Data Availability

The original contributions presented in the study are included in the article/supplementary material. Further inquiries can be directed to the corresponding authors.

## References

[1] Xia C Dong X Li H Cao M Sun D He S . Cancer statistics in China and United States, 2022: profiles, trends, and determinants. Chin Med J (Engl). (2022) 135:584–90. doi: 10.1097/cm9.0000000000002108, PMID: 35143424 PMC8920425

[2] Koshy A . Evolving global etiology of hepatocellular carcinoma (HCC): insights and trends for 2024. J Clin Exp Hepatol. (2025) 15:102406. doi: 10.1016/j.jceh.2024.102406, PMID: 39346785 PMC11426038

[3] Wang T Wang WT . Status quo and development of immunotherapy for hepatocellular carcinoma. Sichuan Da Xue Xue Bao Yi Xue Ban. (2023) 54:692–8. doi: 10.12182/20230560108, PMID: 37248607 PMC10475433

[4] Li Q Cao M Lei L Yang F Li H Yan X . Burden of liver cancer: From epidemiology to prevention. Chin J Cancer Res. (2022) 34:554–66. doi: 10.21147/j.issn.1000-9604.2022.06.02, PMID: 36714347 PMC9829497

[5] Zhang Q He Y Luo N Patel SJ Han Y Gao R . Landscape and dynamics of single immune cells in hepatocellular carcinoma. Cell. (2019) 179:829–45.e20. doi: 10.1016/j.cell.2019.10.003, PMID: 31675496

[6] Gu Y Zhang Z Huang H Zhu W Liu H Zhang R . The dual role of CXCL9/SPP1 polarized tumor-associated macrophages in modulating anti-tumor immunity in hepatocellular carcinoma. Front Immunol. (2025) 16:1528103. doi: 10.3389/fimmu.2025.1528103, PMID: 40230843 PMC11994707

[7] Xin X Cheng X Zeng F Xu Q Hou L . The role of TGF-β/SMAD signaling in hepatocellular carcinoma: from mechanism to therapy and prognosis. Int J Biol Sci. (2024) 20:1436–51. doi: 10.7150/ijbs.89568, PMID: 38385079 PMC10878151

[8] Gungor MZ Uysal M Senturk S . The bright and the dark side of TGF-β signaling in hepatocellular carcinoma: mechanisms, dysregulation, and therapeutic implications. Cancers (Basel). (2022) 14. doi: 10.3390/cancers14040940, PMID: 35205692 PMC8870127

[9] Aguilar-Chaparro MA Rivera-Pineda SA Hernández-Galdámez HV Ríos-Castro E Garibay-Cerdenares OL Piña-Vázquez C . Transforming growth factor-β Modulates cancer stem cell traits on CD44 subpopulations in hepatocellular carcinoma. J Cell Biochem. (2025) 126:e70003. doi: 10.1002/jcb.70003, PMID: 39943801 PMC11833284

[10] Honda CK Kurozumi S Fujii T Pourquier D Khellaf L Boissiere F . Cancer-associated fibroblast spatial heterogeneity and EMILIN1 expression in the tumor microenvironment modulate TGF-β activity and CD8(+) T-cell infiltration in breast cancer. Theranostics. (2024) 14:1873–85. doi: 10.7150/thno.90627, PMID: 38505604 PMC10945331

[11] Arafeh R Shibue T Dempster JM Hahn WC Vazquez F . The present and future of the Cancer Dependency Map. Nat Rev Cancer. (2025) 25:59–73. doi: 10.1038/s41568-024-00763-x, PMID: 39468210

[12] Satija R Farrell JA Gennert D Schier AF Regev A . Spatial reconstruction of single-cell gene expression data. Nat Biotechnol. (2015) 33:495–502. doi: 10.1038/nbt.3192, PMID: 25867923 PMC4430369

[13] Hu C Li T Xu Y Zhang X Li F Bai J . CellMarker 2.0: an updated database of manually curated cell markers in human/mouse and web tools based on scRNA-seq data. Nucleic Acids Res. (2023) 51:D870–d6. doi: 10.1093/nar/gkac947, PMID: 36300619 PMC9825416

[14] Aran D Looney AP Liu L Wu E Fong V Hsu A . Reference-based analysis of lung single-cell sequencing reveals a transitional profibrotic macrophage. Nat Immunol. (2019) 20:163–72. doi: 10.1038/s41590-018-0276-y, PMID: 30643263 PMC6340744

[15] Ru B Huang J Zhang Y Aldape K Jiang P . Estimation of cell lineages in tumors from spatial transcriptomics data. Nat Commun. (2023) 14:568. doi: 10.1038/s41467-023-36062-6, PMID: 36732531 PMC9895078

[16] Larsson L Franzén L Ståhl PL Lundeberg J . Semla: a versatile toolkit for spatially resolved transcriptomics analysis and visualization. Bioinformatics. (2023) 39. doi: 10.1093/bioinformatics/btad626, PMID: 37846051 PMC10597621

[17] Jin S Plikus MV Nie Q . CellChat for systematic analysis of cell-cell communication from single-cell transcriptomics. Nat Protoc. (2025) 20:180–219. doi: 10.1038/s41596-024-01045-4, PMID: 39289562

[18] Fan C Chen F Chen Y Huang L Wang M Liu Y . irGSEA: the integration of single-cell rank-based gene set enrichment analysis. Brief Bioinform. (2024) 25. doi: 10.1093/bib/bbae243, PMID: 38801700 PMC11129768

[19] Hanahan D Michielin O Pittet MJ . Convergent inducers and effectors of T cell paralysis in the tumour microenvironment. Nat Rev Cancer. (2025) 25:41–58. doi: 10.1038/s41568-024-00761-z, PMID: 39448877

[20] Massagué J Sheppard D . TGF-β signaling in health and disease. Cell. (2023) 186:4007–37. doi: 10.1016/j.cell.2023.07.036, PMID: 37714133 PMC10772989

[21] Salaün C Leroy C Rousseau A Boitez V Beck L Friedlander G . Identification of a novel transport-independent function of PiT1/SLC20A1 in the regulation of TNF-induced apoptosis. J Biol Chem. (2010) 285:34408–18. doi: 10.1074/jbc.M110.130989, PMID: 20817733 PMC2966055

[22] Mousseau G Préault N Souquere S Bireau C Cassonnet P Bacquin A . Sodium-dependent phosphate transporter PiT1/SLC20A1 as the receptor for the endogenous retroviral envelope syncytin-B involved in mouse placenta formation. J Virol. (2024) 98:e0091524. doi: 10.1128/jvi.00915-24, PMID: 39287391 PMC11495048

[23] Beck L Leroy C Beck-Cormier S Forand A Salaün C Paris N . The phosphate transporter PiT1 (Slc20a1) revealed as a new essential gene for mouse liver development. PloS One. (2010) 5:e9148. doi: 10.1371/journal.pone.0009148, PMID: 20161774 PMC2818845

[24] Qian X Jin M Bei Y Zhou C Fang S Liu K . SLC20A1 is a prospective prognostic and therapy response predictive biomarker in head and neck squamous cell carcinoma. Aging (Albany NY). (2024) 16:4423–44. doi: 10.18632/aging.205597, PMID: 38412319 PMC10968711

[25] Forand A Koumakis E Rousseau A Sassier Y Journe C Merlin JF . Disruption of the phosphate transporter Pit1 in hepatocytes improves glucose metabolism and insulin signaling by modulating the USP7/IRS1 interaction. Cell Rep. (2016) 16:2736–48. doi: 10.1016/j.celrep.2016.08.012, PMID: 27568561

[26] Peng D Fu M Wang M Wei Y Wei X . Targeting TGF-β signal transduction for fibrosis and cancer therapy. Mol Cancer. (2022) 21:104. doi: 10.1186/s12943-022-01569-x, PMID: 35461253 PMC9033932

[27] Wu L Yan J Bai Y Chen F Zou X Xu J . An invasive zone in human liver cancer identified by Stereo-seq promotes hepatocyte-tumor cell crosstalk, local immunosuppression and tumor progression. Cell Res. (2023) 33:585–603. doi: 10.1038/s41422-023-00831-1, PMID: 37337030 PMC10397313

[28] Maestri E Kedei N Khatib S Forgues M Ylaya K Hewitt SM . Spatial proximity of tumor-immune interactions predicts patient outcome in hepatocellular carcinoma. Hepatology. (2024) 79:768–79. doi: 10.1097/hep.0000000000000600, PMID: 37725716 PMC10948323

[29] Alqurashi YE Al-Hetty H Ramaiah P Fazaa AH Jalil AT Alsaikhan F . Harnessing function of EMT in hepatocellular carcinoma: From biological view to nanotechnological standpoint. Environ Res. (2023) 227:115683. doi: 10.1016/j.envres.2023.115683, PMID: 36933639

